# Case Report: Presymptomatic risdiplam in preterm monozygotic twins with co-occurring spinal muscular atrophy and tuberous sclerosis complex

**DOI:** 10.3389/fgene.2026.1851666

**Published:** 2026-09-07

**Authors:** Xiao-fang Zhang, Yan-juan Wang, Si-xiu Li, Ping Liu, Wen-Guang Hu

**Affiliations:** Department of Pediatric Neurology, Chengdu Women’s and Children’s Central Hospital, School of Medicine, University of Electronic Science and Technology of China, Chengdu, China

**Keywords:** monozygotic twins, presymptomatic, risdiplam, spinal muscular atrophy, tuberous sclerosis complex

## Abstract

**Background:**

Spinal muscular atrophy (SMA) and tuberous sclerosis complex (TSC) are both rare genetic disorders, and their co-occurrence is expected to be exceptionally uncommon. Although risdiplam has demonstrated efficacy in SMA, evidence regarding its use in preterm infants with complex genetic comorbidities remains limited.

**Results:**

We report preterm monozygotic twins born at 32+5 weeks of gestation with genetically confirmed SMA caused by homozygous deletion of SMN1 exons 7–8, with two copies of SMN2. During baseline evaluation prior to presymptomatic treatment, brain magnetic resonance imaging revealed incidental abnormalities, including cortical dysplasia and subependymal nodules, which prompted further genetic testing and led to the diagnosis of TSC with a TSC1 c.1041G>A variant. Presymptomatic risdiplam was initiated at a corrected gestational age of 38+5 weeks. Both twins showed marked improvement in motor function. By 12 months of age, they had achieved independent rolling, unsupported sitting, crawling, and pulling to stand, accompanied by substantial improvements in CHOP INTEND and HINE-2 scores. Risdiplam was well tolerated, and no commonly reported adverse events were observed during follow-up.

**Conclusion:**

This report describes the first documented co-occurrence of SMA and TSC in preterm monozygotic twins and suggests that presymptomatic risdiplam may be effective and well tolerated in this complex clinical setting over 1 year of follow-up. These findings also highlight the importance of comprehensive genetic evaluation in infants with SMA who present with atypical neuroimaging or neurological features. Longer-term follow-up is needed to clarify safety, efficacy, and optimal integrated management strategies in patients with complex genetic comorbidities.

## Introduction

Spinal muscular atrophy (SMA) is an autosomal recessive neuromuscular disorder and the most common genetic cause of infant death. Approximately 95% of SMA cases stem from homozygous deletions (95%) or compound heterozygous mutations (5%) in exon 7 or exons 7 and 8 of the Survival Motor Neuron 1 (SMN1) gene at 5q13.2. (Kolb and Kissel, 2015), resulting in a severe deficiency of functional SMN protein. The paralogous SMN2 gene produces only a limited amount of full-length SMN protein (approximately 5%–10%) because a C>T transition in exon 7 promotes alternative splicing, leading predominantly to truncated and unstable transcripts (Lorson et al., 1999; Monani et al., 1999). Clinically, SMA encompasses a broad phenotypic spectrum and is traditionally classified into types 0–4 according to age at onset and the highest motor milestone achieved (Wirth et al., 2020). Types 1–3 usually present in childhood with progressive muscle weakness and atrophy, often accompanied by respiratory, gastrointestinal, and skeletal complications that substantially impair survival and quality of life. SMN2 copy number inversely correlates with disease severity: two copies predict type 1 (79% probability), three copies predict type 2 (54%), and four copies predict milder type 3 (88%) (Calucho et al., 2018).

Tuberous sclerosis complex (TSC), another childhood-onset genetic disorder, is inherited in an autosomal dominant manner and is caused by pathogenic variants in either TSC1 on chromosome 9q34.3 or TSC2 on chromosome 16p13.3. (Northrup et al., 2013; Northrup et al., 2021). TSC is characterized by the formation of hamartomas in multiple organs, including the brain, skin, eyes, heart, kidneys, liver, and lungs, and by a broad range of neurological and neuropsychiatric manifestations, such as epilepsy, cognitive impairment, learning difficulties, and autism spectrum disorder. Although SMA and TSC are genetically and clinically distinct disorders, their coexistence in a single patient is exceptionally rare and may obscure the clinical picture, posing challenges for diagnosis, management, and genetic counseling.

Risdiplam, one of the three currently available disease-modifying therapies for SMA, is an orally administered SMN2 pre-mRNA splicing modifier that increases the production of functional SMN protein. Its safety and efficacy have been demonstrated in symptomatic patients with SMA types 1–3 across both pediatric and adult populations (Mercuri et al., 2022; Masson et al., 2022; Saito et al., 2025; Baranello et al., 2021). Growing evidence highlights the critical importance of presymptomatic intervention. For instance, Finkel et al. reported that infants who received risdiplam before the onset of clinical symptoms (≤6 weeks of age) achieved better motor and survival outcomes at 12 and 24 months than those observed in natural-history cohorts (Finkel et al., 2025). Similarly, observational data reported by Cooper et al. further suggest that treatment initiated before symptom onset is associated with more favorable outcomes than treatment started after clinical manifestation (Cooper et al., 2024).

Nevertheless, the available evidence on risdiplam has been derived predominantly from studies involving term neonates and infants. Data regarding its safety, pharmacokinetics, and efficacy in preterm infants remain extremely limited, which is of particular concern given their organ immaturity, frequent comorbidities, and distinct pharmacokinetic profiles. Moreover, no studies to date have described the use of risdiplam in infants with coexisting SMA and TSC, particularly in those harboring pathogenic TSC1 variants. The overlapping pathophysiological mechanisms, potential treatment-related interactions, and multisystem involvement may pose unique therapeutic challenges in this highly specific population.

Here, we report what is, to our knowledge, the first case in China of monozygotic twins born at 32+5 weeks of gestation with genetically confirmed 5q SMA, characterized by homozygous deletion of SMN1 exons 7 and 8 and an SMN2 copy number of 2, together with a pathogenic TSC1 variant (c.1041G>A, p.Trp347). Risdiplam was initiated at 6 weeks after birth, corresponding to a corrected gestational age of 38+5 weeks, before the onset of SMA-related symptoms. This report aims to provide preliminary clinical evidence for presymptomatic risdiplam treatment in Chinese preterm infants, explore its safety and potential efficacy in the setting of coexisting SMA and a TSC1 variant, and offer practical insights into the precise diagnosis and management of complex genetic comorbidities.

## Case report

This report describes female monozygotic twins, established by prenatal ultrasound and obstetric records showing monochorionic-diamniotic placentation, hereafter referred to as Twin A and Twin B. They were born vaginally at 32+5 weeks of gestation. Newborn screening using quantitative polymerase chain reaction (qPCR) and multiplex ligation-dependent probe amplification (MLPA) identified a homozygous deletion of exons 7 and 8 in the SMN1 gene, with an SMN2 copy number of 2, in both infants. These findings confirmed the diagnosis of 5q spinal muscular atrophy (5q SMA). They were transferred to our department for further evaluation and management on 19 November 2024, at a corrected gestational age of 38+5 weeks (postnatal age, 1 month and 9 days). On admission, neurological examination showed preserved antigravity movements in all limbs, normal muscle tone, symmetric bilateral patellar and Achilles reflexes, and no tongue fasciculations during spontaneous crying. Baseline motor assessment revealed comparable findings in the two twins, with each infant having a Children’s Hospital of Philadelphia Infant Test of Neuromuscular Disorders (CHOP INTEND) score of 37, a Hammersmith Infant Neurological Examination, Section 2 (HINE-2), score of 3, and normal compound muscle action potential amplitudes in the lower extremities.

**FIGURE 1:**
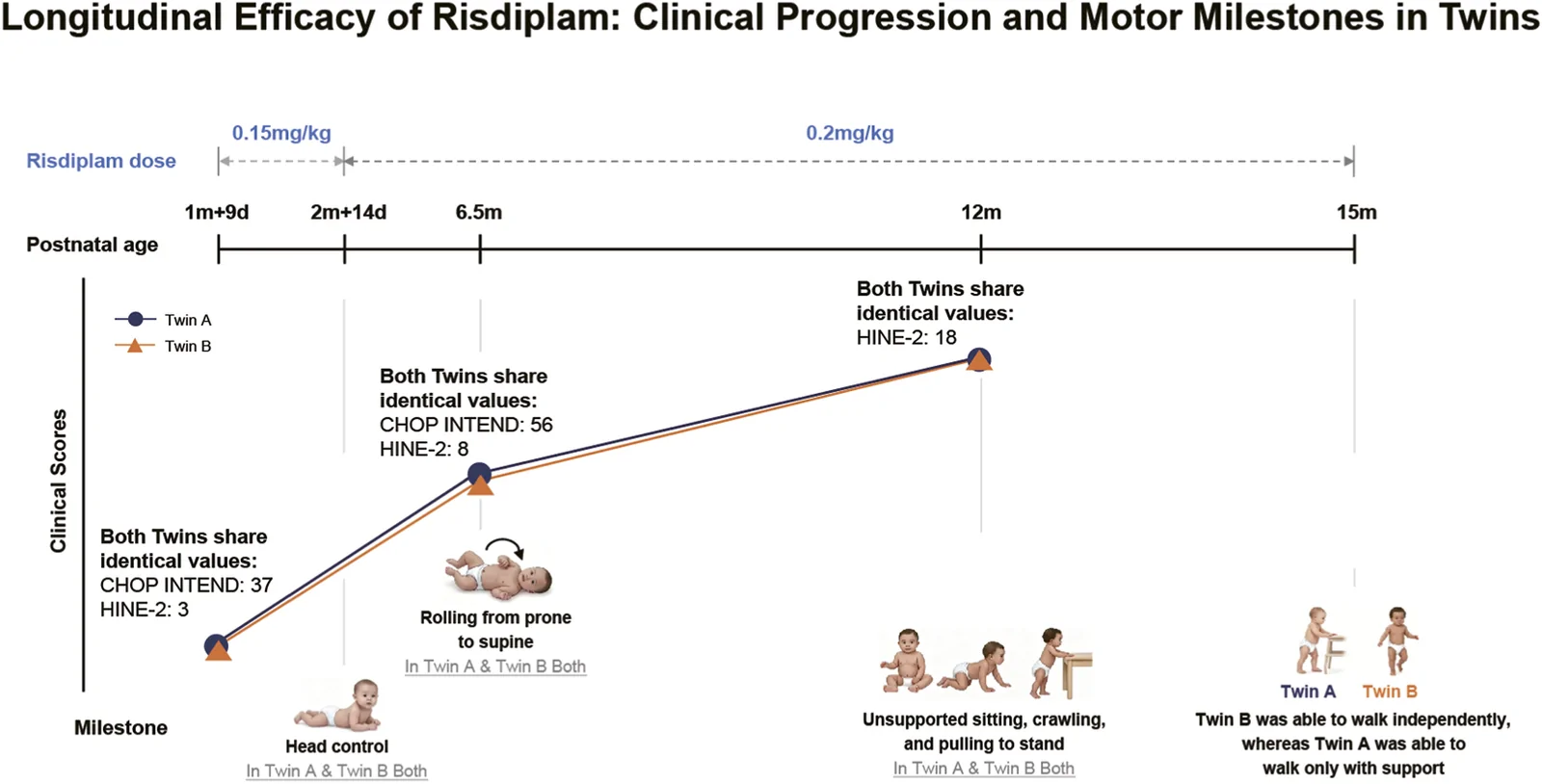
Longitudinal motor assessments and major motor milestones in the twins before and during risdiplam treatment.

Presymptomatic treatment with risdiplam was initiated on 21 November 2024, at the recommended dose of 0.15 mg/kg/day. In accordance with the FDA-approved prescribing information for risdiplam, the dose was increased to 0.2 mg/kg/day at postnatal day 75 and maintained throughout the 15-month follow-up period. No treatment interruptions or missed doses occurred, with 100% adherence confirmed by pharmacy dispensing records and caregiver logs. Following treatment initiation, both infants showed sustained improvement in motor function. At approximately 1 month after treatment initiation, both twins achieved head control and visual tracking. By approximately 6.5 months of age, both had achieved rolling from prone to supine, with CHOP INTEND and HINE-2 scores improving to 56 and 8, respectively. At the subsequent follow-up visit on 22 October 2025 (approximately 12 months of age), the HINE-2 score had further increased to 18 in both infants, and both had achieved. Motor milestones achieved at that time included independent rolling, unsupported sitting, crawling, and pulling to stand. Both infants maintained adequate swallowing for full oral feeding and stable respiratory function without the need for ventilatory support. Neurological examination continued to demonstrate preserved antigravity movements in all limbs, normal muscle tone, and symmetric 2+ patellar and Achilles reflexes. Although the overall treatment response was favorable in both infants, mild divergence in later motor development became evident during continued follow-up. By 15 months of age, Twin B was able to walk independently, whereas Twin A was able to walk only with support. To clarify the longitudinal motor trajectories of the two patients, we have separately presented serial motor assessments and major motor milestones for each twin in Figure 1. Risdiplam was well tolerated throughout follow-up. Except for one episode of respiratory infection in Twin A that was considered unrelated to treatment, neither infant experienced common risdiplam-associated adverse events, including fever, diarrhea, rash, oral ulceration, arthralgia, or urinary tract infection.

During the neonatal period, both twins were also diagnosed with congenital hypothyroidism on the basis of elevated thyrotropin levels. This was effectively controlled with levothyroxine sodium replacement, and thyroid function remained within the normal range throughout follow-up. In addition, baseline brain MRI, performed as part of the initial clinical evaluation, incidentally revealed central nervous system abnormalities in both infants. Twin A showed a polymicrogyria-like cortical malformation (cortical dysplasia) involving the left perisylvian region and occipital cortex, together with a small left subependymal nodule (SEN), whereas Twin B had a small right SEN adjacent to the body of the lateral ventricle.

**FIGURE 2:**
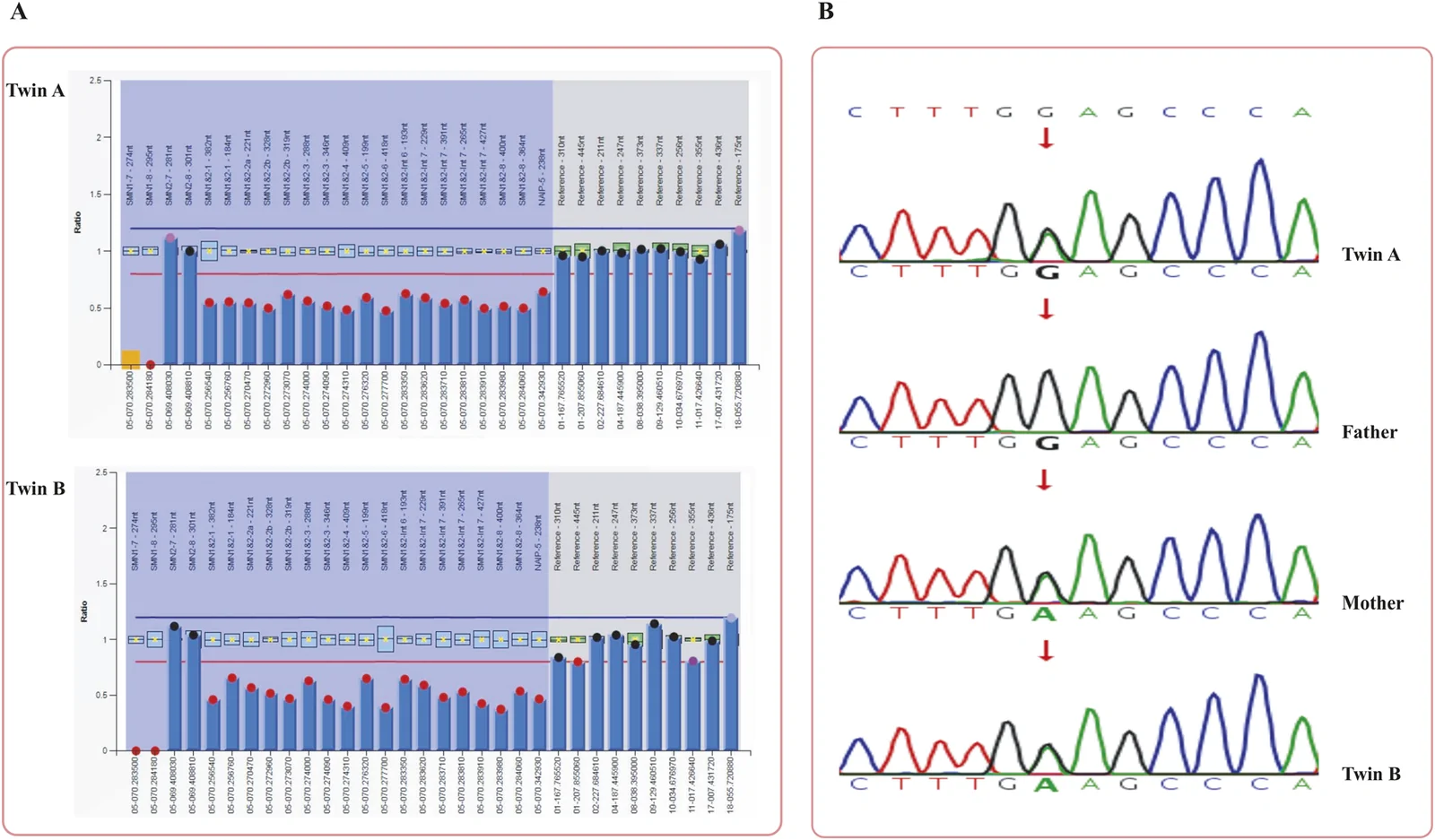
Genetic analyses of the preterm twins with co-occurring SMA and TSC. **(A)** MLPA analysis demonstrating homozygous deletion of SMN1 exons 7 and 8 in both twins. **(B)** Sanger sequencing chromatograms confirming the heterozygous TSC1 variant (c.1041G>A, p.Trp347) and its segregation within the family.

These findings prompted genetic evaluation for tuberous sclerosis complex. Whole-exome sequencing identified the same heterozygous pathogenic TSC1 variant (NM_000368.5:c.1041G>A, p.Trp347Ter; chr9:132911102G>A) in both twins (Figure 2), inherited maternally. This nonsense variant was absent in population databases (gnomAD/ExAC/1000G) and classified as Pathogenic in ClinVar, fulfilling ACMG/AMP criteria (PVS1, PP4, PM2_Supporting). According to international diagnostic consensus, identification of this pathogenic variant establishes the diagnosis of tuberous sclerosis complex type 1 (OMIM #191100), even without fully developed clinical manifestations (Northrup et al., 2021). Familial segregation analysis demonstrated maternal inheritance of this variant. Physical examination revealed no hypomelanotic macules in the mother, and brain MRI showed no abnormalities.

**FIGURE 3:**
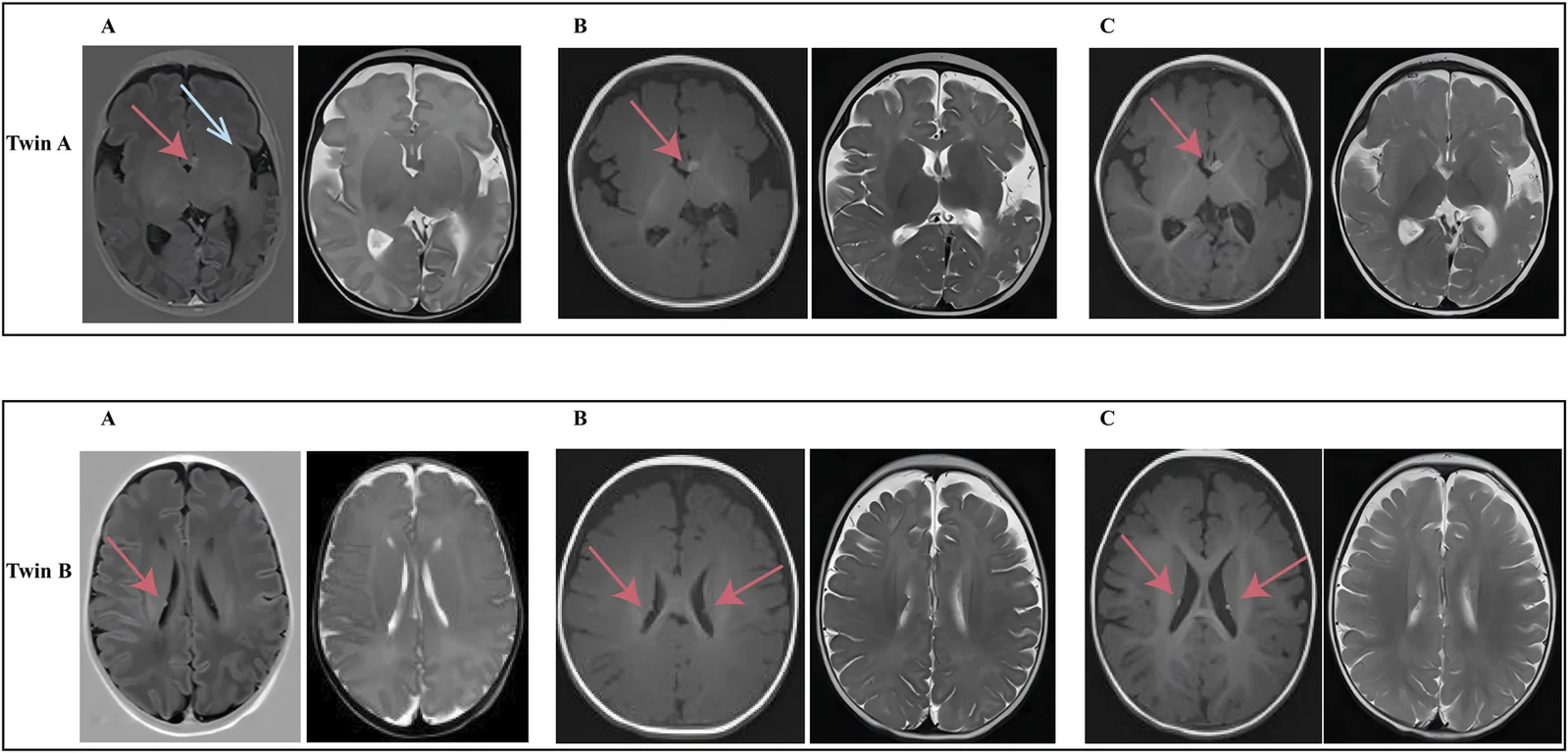
Serial brain MRI findings in a preterm twin with co-occurring SMA and TSC. **(A)** Baseline MRI at 6 weeks of age; **(B)** Follow-up MRI at 6.5 months of age; **(C)** Follow-up MRI at 12 months of age. For each patient and time point, the left and right images show T1-weighted and T2-weighted sequences, respectively. In Twin A, the blue arrow indicates a stable polymicrogyria-like cortical malformation, whereas the red arrows indicate left subependymal nodules (SENs), which increased slightly in number and size at 6.5 months and remained visible at 12 months. In Twin B, red arrows indicate a right SEN at baseline and a newly identified left SEN at 6.5 months; bilateral SENs remained stable at 12 months.

Subsequent systematic evaluation for TSC demonstrated that Twin A exhibited stable cortical malformations on serial MRI, while the left SENs showed slight interval increase in size and number at 6.5 months with persistence at 12 months. Twin A developed hypomelanotic macules. Conversely, Twin B demonstrated a new left SEN at 6.5 months, with stable bilateral SENs at 12-month follow-up and no cutaneous manifestations (Figure 3). Serial ultrasonography detected no cardiac rhabdomyomas or renal angiomyolipomas in either infant. Neonatal electroencephalography was normal in both twins. Serial follow-up EEGs were not performed because neither twin exhibited clinically apparent seizures during follow-up. In addition, prolonged EEG recordings were difficult to obtain because of limited cooperation from the patients. Nevertheless, the parents noted subtle neurological differences between the twins: compared with Twin B, Twin A exhibited mild right-sided motor incoordination and slower responsiveness in daily interactions.

## Discussion

Spinal muscular atrophy has traditionally been characterized as a motor neuron disease. However, emerging evidence has documented cognitive impairment and neuroimaging abnormalities in a subset of infants with SMA type 0 or 1 (Giannotta et al., 2024; Mendonça et al., 2019). Based on these observations, we initiated presymptomatic risdiplam treatment, with baseline brain MRI screening performed at treatment initiation. Unexpectedly, brain MRI revealed cortical dysplasia and subependymal nodules, which prompted further genetic testing and ultimately led to the diagnosis of TSC. The incidence of SMA is approximately 1 in 11,000 live births, with a carrier frequency ranging from 1 in 40 to 1 in 70, making it the most common monogenic cause of infant mortality (Verhaart et al., 2017). Tuberous sclerosis complex has an estimated incidence of approximately 1 in 6,760 to 1 in 13,520 live births (Ebrahimi-Fakhari et al., 2018). Given that both are rare genetic disorders, their co-occurrence would be expected to be exceptionally uncommon. To the best of our knowledge after quick literature review, this is the first reported case worldwide. In addition, several cases of SMA co-occurring with other pathogenic gene variants have been reported, as summarized in Table 1 (Xia et al., 2021; Ma et al., 2024; Pandey et al., 2011; Voutoufianakis et al., 2007; Jedrzejowska et al., 2008). These findings underscore the importance of comprehensive genetic and metabolic evaluation, combined with multimodal molecular testing, in infants with congenital abnormalities, thereby reducing the risk of missed or delayed diagnosis of coexisting genetic disorders. In particular, when a patient’s clinical course deviates from the expected trajectory of a single disorder, the possibility of dual molecular diagnoses should be considered and further testing pursued.

**TABLE 1:** Cases with co-occurrence of additional genetic disorders in individuals with SMA.

Source (year)	Co-occurring genetic disorder	Mutated gene
1. Xia Y (2021)	DMD	*DMD* (XR)
2. Ma K (2024)	Phenylketonuria	*PAH* (AR)
3. Pandey R (2010)	Central core myopathy	*RYR1* (AD)
4. Voutoufianakis S (2007)	Rett syndrome	*MECP2* (XD)
5. Jedrzejowska M (2008)	Charcot-Marie-Tooth	*PMP22* (AD)

AR, autosomal recessive; AD, autosomal dominant; XR, X-linked recessive inheritance; XD X-linked dominant inheritance; DMD, Duchenne Muscular Dystrophy; PAH: phenylalanine hydroxylase; RYR1 Ryanodine Receptor 1; MECP2 Methyl-CpG, Binding Protein 2; PMP22 Peripheral Myelin Protein 22.

Another notable finding in this family was the phenotypic variability among individuals carrying the same pathogenic TSC1 variant. Although both twins and their mother harbored this variant, their clinical manifestations differed considerably. Twin A developed progressive intracranial abnormalities and hypomelanotic macules, whereas Twin B showed a milder and more stable clinical course.

Phenotypic heterogeneity has been described in both familial and monozygotic twin cases of TSC. Wang et al. (2018) reported marked intrafamilial clinical variability in a Chinese family carrying a heterozygous TSC2 variant. Similarly, Xiong et al. (2022) described discordant prenatal manifestations in monozygotic twins with a TSC1 mutation, involving differences in the onset and progression of cardiac rhabdomyomas and central nervous system lesions. Fan et al. (2026) also reported substantial clinical differences between monozygotic twin sisters with TSC-associated lymphangioleiomyomatosis carrying the same TSC2 variant. These reports indicate that clinical differences in TSC may arise as early as the prenatal period and may not be determined solely by the underlying germline TSC1 or TSC2 variant.

The mechanisms underlying phenotypic variability in TSC remain incompletely understood. Liang et al. identified alterations in mTOR pathway-related genes that were associated with mild or severe phenotypes among affected individuals within the same families, suggesting a potential role for genetic modifiers (Liang et al., 2017). In addition, emerging evidence suggests that epigenetic mechanisms, including DNA methylation, histone modifications, and dysregulation of non-coding RNAs, may contribute to phenotypic variation in TSC (Hadjigavriel et al., 2025). These factors may be relevant to the clinical differences observed between the twins in the present study.

The co-occurrence of these two disorders not only poses diagnostic challenges but also raises intriguing questions about potential pathological interactions at the molecular level. In tuberous sclerosis complex, loss of function of the TSC1/TSC2 complex leads to constitutive activation of the RHEB/mTORC1 signaling axis. This hyperactivation disrupts autophagic flux by inhibiting autophagosome formation and lysosomal biogenesis through phosphorylation-dependent suppression of the ULK1 complex and the transcription factor TFEB (Wullschleger et al., 2006; Luo et al., 2022). This autophagic deficiency promotes aberrant protein aggregation and mitochondrial dysfunction, thereby contributing to neurodevelopmental abnormalities, cortical tuber formation, and epileptogenesis (MacKeigan and Krueger, 2015; Previtali et al., 2023). Studies in spinal muscular atrophy (SMA) have revealed cell type-specific alterations in autophagy: in motor neurons, reduced mTOR phosphorylation is associated with autophagosome accumulation and impaired degradation, whereas in muscle and fibroblasts, increased mTOR phosphorylation or reduced total mTOR expression may inhibit autophagosome formation (Sansa et al., 2021; Rashid and Dimitriadi, 2024). Notably, although TSC is characterized by mTOR hyperactivation and SMA motor neurons by relative mTOR inhibition, both conditions may converge on neuronal injury through disruption of autophagic homeostasis. The potential pathological crosstalk between TSC and SMA in patients with both conditions warrants further investigation.

We report preterm monozygotic twins born at 32+5 weeks of gestation with genetically confirmed SMA and two copies of SMN2, a genotype predictive of SMA type 1. Following presymptomatic initiation of risdiplam, both infants achieved rolling from prone to supine by approximately 6.5 months of age. By 12 months of age, both had achieved independent rolling, unsupported sitting, crawling, and pulling to stand. These outcomes contrast with natural history data showing that approximately 79% of untreated infants with two SMN2 copies develop SMA type 1 and do not achieve independent sitting (Calucho et al., 2018). These outcomes are consistent with the favorable effects of risdiplam in presymptomatic SMA, as reported by Finkel et al. (2025). Although commonly reported adverse events associated with risdiplam include diarrhea (52%), asthenia (48%), fatigue (45%), nausea (38%), and pneumonia (32%) 30, neither infant in the present report experienced these typical adverse events. To our knowledge, this is the first reported use of risdiplam in preterm infants with SMA and coexisting TSC, and these observations may provide preliminary evidence to inform the clinical management of this unique patient population.

In conclusion, this report describes the first documented co-occurrence of SMA and TSC in preterm monozygotic twins. These findings underscore the importance of comprehensive genetic evaluation in patients with SMA who exhibit atypical clinical features. Presymptomatic initiation of risdiplam at a corrected gestational age of 38+5 weeks was associated with sustained improvement in motor function. Collectively, these observations are consistent with a potential benefit of early SMN-targeted therapy in this complex preterm genetic context and suggest favorable tolerability of risdiplam. Further studies are warranted to evaluate the long-term safety and functional outcomes of early risdiplam intervention in patients with complex genetic comorbidities.

This study has several limitations. First, because of the limited duration of follow-up, the long-term efficacy of risdiplam and its potential interaction with the TSC disease course remain unclear. Second, treatment was directed exclusively at SMA, and no TSC-specific therapy was administered. Consequently, the potential reciprocal influence of these two disorders on their respective natural histories and treatment responses could not be determined. Third, although neonatal electroencephalography was normal in both twins, serial follow-up EEG data were not available, particularly for Twin A, who showed cortical dysplasia and progression of intracranial lesions. This limited our ability to more comprehensively assess subclinical epileptiform activity and longitudinal neurophysiological changes. Future studies with longer follow-up are needed to clarify this complex interplay and to optimize integrated management strategies.

## Data Availability

The datasets presented in this article are not readily available because of ethical and privacy restrictions. Requests to access the datasets should be directed to the corresponding author.
